# Supplementary material for: Are carbon emissions trading and green financial instruments synergistic? -Comprehensive quantitative research based on content analysis
Source: PLoS One. 2024 Mar 7;19(3):e0298601. doi: 10.1371/journal.pone.0298601 (PMC10919601; doi:10.1371/journal.pone.0298601)
Supplement: S1 Data — (ZIP) [file pone.0298601.s001.zip › Data/地级市Z_F工作报告.zip!/░▓╗╒/░÷▓║╩╨/└┤╫╘╣1⁄2╓┌║┼í╛┬φ┐╦╩2╛▌═°í┐_2009.docx]

各位代表：

    现在，我代表市人民政府向大会报告工作，请予审议，并请市政协委员和其他列席人员提出意见。

    一、奋力拼搏进取，2008年各项目标任务基本实现

    2008年是很不寻常、很不平凡的一年，也是我市改革发展进程中非常重要的一年。一年来，在中共蚌埠市委的坚强领导下，全市上下坚持以科学发展观为统领，积极应对雨雪冰冻灾害、国际金融危机等严峻困难和挑战，按照市十四届人大一次、二次会议确定的各项目标任务和工作部署，锐意进取，扎实工作，基本完成了各项目标任务，经济社会保持了良好的发展势头。预计，全年完成地区生产总值486亿元，同比增长11%，其中，一、二、三次产业增加值分别增长6%、14.5%、10%。财政总收入65.7亿元，增长15%，其中，地方财政收入27.1亿元，增长20%。固定资产投资完成250亿元，增长26.5%。进出口总额3.9亿美元，增长22%。实际利用外资2.32亿美元、引进内资180亿元，分别增长15%、53.8%。社会消费品零售总额189亿元，增长21.2%。城镇居民人均可支配收入12700元、农民人均纯收入4160元，分别增长11%、15%。万元地区生产总值能耗降低4.4%。

    (一)工业经济稳步发展。扎实推进工业强市战略，加大投入，加强调节，强化服务，努力保持工业经济平稳较快增长。规模以上工业实现增加值137亿元，增长18%。着力培育骨干企业，销售收入亿元以上企业超50户，新增规模以上企业112户。加快项目建设步伐，实施千万元以上重点项目200个。丰原生化燃料乙醇装置改造、华润10万吨啤酒扩建、八一化工5000吨愈创木酚等50个项目竣工投产。昊方500万套汽车空调电磁离合器技改、华益年产400万片抗反射膜玻璃、五河凯迪秸秆发电等50个项目开工建设。积极推进园区建设，省级开发区新增入园工业项目107个。企业改制和重组工作取得新进展，全面完成18家市属国有企业破产案件收尾工作，华皖碳纤维、丰原集团等企业改制工作稳步推进，广西柳工集团成功重组振冲安利。

    (二)农业和农村经济再上新台阶。实施小麦、水稻、玉米三大粮食生产行动，粮食总产再创新高，达253万吨，增长11.5%，连续五年实现丰产丰收。大力推进农业产业化经营，新增省级龙头企业6家，怀远县被列为省级农业产业化示范县。着力提升农产品质量安全水平，新增无公害农产品、绿色食品、有机食品认证20个，省级以上农业标准化示范区达11个。进一步改善农村生产条件，完成淮干蚌埠至浮山段河道整治、淮北大堤和14座小水库除险加固，芡河、跃进沟、黑泥沟洼地治理工程全面竣工，改造中低产田14万亩，成片造林1.38万亩，新增绿色长廊75公里。畜牧兽医体制改革、集体林权制度改革基本完成。县域经济发展步伐加快，县域地区生产总值、财政收入、固定资产投资总额增速均高于全市平均水平。

    (三)第三产业规模不断扩大。加大城乡商业基础设施建设力度。光彩客运南站、延安路农贸市场、白马商城等建成投入运营。宝龙城市广场、合百新商厦、沃尔玛购物中心建设加快推进。商之都项目开工建设。英国乐购集团进驻蚌埠。"万村千乡"市场工程和"新网工程"稳步推进，新建日用消费品农家店180个。加快发展现代服务业。金融对地方经济的支持力度进一步加大，邮政储蓄银行、怀远农村合作银行挂牌，5家小额贷款公司获批筹建。旅游业发展势头良好，龙子湖风景区、禾泉农庄成功创建国家4A级景区，孙家圩子渡江战役总前委纪念馆(旧址)建成开馆。物流、信息、咨询、中介等现代服务业加快发展。

    (四)招商引资和对外开放取得新成效。坚持把招商引资作为经济工作的重中之重，推进招商引资工作持续升温提速。突出开展"招商引资月"活动，成功举办"第二届中国花鼓灯歌舞节暨蚌埠投资贸易洽谈会"，全年新引进项目340个。对外开放成效明显，外贸出口逆势上扬，全年实现出口3.55亿美元，增长26%。实施企业"走出去"战略，实现对外经济合作营业额7000万美元。深入开展全民创业，大力发展非公有制经济，新增个体工商户9875户、私营企业1586户，非公有制经济占国民经济比重达54%。我市荣获省发展非公有制经济先进市称号。

    (五)固定资产投资增长较快。狠抓投资和项目建设，大力实施"3461"行动计划。投资结构进一步优化。工业投资首次突破100亿元，达120亿元，占全市投资近50%。项目资金落实成效明显。争取开行贷款额度近10亿元，落实国债和专项资金3.1亿元，银企对接活动签约项目累计到位资金63.9亿元。重点项目建设进展顺利。国电蚌埠电厂第一台机组、普乐新能源12兆瓦非晶硅太阳能电池等23个项目已建成或基本建成，京沪高铁蚌埠段、姜桥物流工业园、固镇煤炭资源开发等52个项目实现开工，城市环境综合整治世行项目、水环境综合治理日元项目进入正式实施阶段，国电蚌埠电厂二期、大庆路淮河公路桥、蚌淮高速公路连接线等一批项目前期工作有效推进。

    (六)城市面貌有所改善。城市总体规划通过省政府审批，城市综合交通等5个专业规划编制完成，建成并开放城市规划展示馆。东海大道--解放路立交工程和燕山路东段、解放路南段、迎湖路改造竣工通车，城市西出口、北出口改造基本完工。龙子湖北公园建成开园，张公湖综合治理初现成效。31个棚户区改造逐步展开。大板楼危房改造基本完成。城市管理工作有新的加强，启动城市管理体制改革和数字化城市管理模式试点工作。开展违章占道、饮食摊点群等专项整治，市容市貌持续改善。我市荣获省首届文明城市称号。

    (七)自主创新综合配套改革试验全面启动。经过争取，我市进入合芜蚌自主创新综合配套改革试验区。及时制定出台了实施方案，明确了工作目标和任务，召开了启动试验区建设动员大会。争取省级资金支持1.5亿元，统筹安排市级配套资金，积极培育创新产业体系，增强企业创新能力，申报各类科技计划项目166项，国家玻璃深加工工程技术研究中心和安徽省薄膜太阳能电池工程技术研究中心挂牌，丰原发酵技术国家工程研究中心通过验收。加快高新技术产业发展，高新技术产业技工贸总收入达170亿元，增长21.4%。知识产权保护和专利工作取得新成绩，全年专利申请量达450件，同比增加1倍。着力推进县区科技进步，怀远县被评为全国科技进步先进县。

    (八)环境保护与治理得到加强。严格控制污染物排放总量，完成省政府下达我市工程减排项目12个，化学需氧量排放控制在1.93万吨，二氧化硫排放控制在2.71万吨。三县污水处理厂投入运行，第三污水处理厂建成试运行，第二污水处理厂基建完成。强化监管技防措施，提高重点污染源自动化监控水平。加强饮用水源地保护工作，城市东部供水工程基本完成。

    (九)民生工程扎实推进。18项民生工程进展顺利，全年投入资金8.1亿元，同比增加1倍，承诺的10件实事全面完成。城镇新增就业人员3.6万人，安置下岗失业人员2.2万人。为9.8万名企业离退休人员及时调增并足额发放养老金。发放城乡低保金1.29亿元。新建改建敬老院28所，五保供养对象分散和集中供养标准分别达到1300元和1817元。发放城乡医疗救助金1064万元。新农合实现全覆盖。新建改建乡镇卫生院29所、农村卫生室156所、社区卫生服务中心(站)28个。改造农村中小学危房4万平方米，免除47.3万名学生学杂费和38.7万名学生书本费，补助住宿贫困学生生活费7965万元。发放农村计划生育奖励扶助金121.9万元。启动105个新农村示范村建设，新改造农村公路500公里、农村公路客运站9个，实施渡改桥工程5个。完成淮河行蓄洪区移民迁建3000户。解决12万农村人口安全饮水问题。为36个村配备健身器材。开工建设经济适用房15.5万平方米和廉租房3.52万平方米，8561户困难家庭享受到廉租住房租赁补贴。顺利推行公交IC卡消费管理系统，新开辟公交线路2条、站点64个。建设改造3个垃圾中转站、25座城市公厕，安装路灯4805套。

    (十)各项社会事业协调发展。调整市区义务教育管理体制，平稳完成市属19所中小学下划工作，安徽粮食经济技师学院和蚌埠技师学院筹建通过省专家组评估。卫生基础设施建设全面加强，公共卫生应急体系进一步完善，第三人民医院门、急诊改扩建首期工程竣工。花鼓灯舞蹈《鼓乡情韵》成功进入奥运会开幕前展演，双墩1号墓、禹墟和垓下古战场考古挖掘取得重大新发现。龙湖体育馆主体工程完工，市体育场馆维修改造工程进展顺利，全民健身运动蓬勃开展。广播电视信息数据平台建设全面完成。食品药品安全监管和市场价格监管扎实有效。积极支持汶川特大地震灾区抗震救灾工作，"刘氏四兄弟"被命名为"全国抗震救灾英模"。高度重视安全生产，继续加强信访工作，强化社会治安综合治理，社会保持和谐稳定。妇女、儿童、老年人、残疾人权益得到保障。人事、统计、档案、外事、侨务、气象、地震、人防、地方志、政府法制、民族宗教、爱国卫生、新闻出版、社会科学研究、人口与计划生育、国防和后备力量建设等工作，都取得新的成绩。

    2008年，我们坚持依法行政，自觉接受人大法律监督和政协民主监督，办理人大代表议案7件、建议121件，政协提案495件。认真执行政府信息公开条例，深入推进政务公开和电子政务。政风行风热线、政风行风面对面、市长热线等反映社情民意的桥梁作用进一步发挥。手足口病防治取得显著成效，妥善处置婴幼儿问题奶粉事件，突发事件应急处置能力进一步增强。扎实开展反腐倡廉制度建设推进年活动，加大行政监察和审计监督力度，严肃查处一批违法违纪案件。

    在这里需要特别指出的是，去年下半年以来，面对突如其来的国际金融危机和严峻的宏观经济形势，我们审时度势，积极应对，认真贯彻国家宏观调控政策，及时出台了促进经济平稳较快增长的12条意见等一系列措施，努力增加有效投资，扩大消费需求，促进经济增长。积极做好扩大内需争取项目工作，共谋划140个项目，向上申报项目80个(类)、资金9亿元。加强经济运行调节，开展工业、商业、农业调研，积极为企业解决煤电油运和资金紧张等问题，向75个重点园区、企业、项目选派93名服务人员。进一步清理行政审批项目，降低行政事业性收费，尽最大可能为企业解困松绑，为企业发展营造宽松环境。通过采取积极有效的应对措施，避免了经济出现较大波动，全市经济社会保持平稳较快发展。

    各位代表！过去的一年，我们走过了不平凡的历程，取得了新的发展成就，成绩来之不易。这是中共蚌埠市委正确领导的结果，是全市人民艰苦努力的结果。在此，我谨代表市人民政府，向全市各族人民，向广大工人、农民、知识分子、干部和解放军指战员、武警官兵、政法干警，向国家和省驻蚌单位，向各民主党派、工商联、各人民团体和其他各界人士，向人大代表、政协委员，向离退休老同志，向在蚌创业的境内外投资者和建设者，向所有关心、支持和参与蚌埠现代化建设的海内外朋友，表示崇高的敬意和衷心的感谢！

    在肯定成绩的同时，我们也清醒地看到，发展中仍然存在一些矛盾和问题。经济运行下滑的压力加大，全市经济总量增速减缓；经济结构还不够优化，产业格局急需加快调整和升级；经济发展方式有待进一步转变，加快发展与节约资源、保护环境的矛盾日益突出；发展后劲和活力不强，投资持续增长的基础不稳固，带动性强的重大项目不多；城镇化水平提高不快，改善民生、城乡统筹以及和谐社会建设压力仍然较大；政府职能转变还不到位，行政执行力和服务水平还需要继续提高等等。对此，我们将高度重视，采取切实有效措施，认真加以解决。

    二、牢牢把握宏观形势，进一步坚定加快发展的信心

    2009年是中华人民共和国成立60周年，也是推进"十一五"规划顺利实施的关键一年。纵观今年发展形势，我们面临的仍然是一个机遇与挑战并存，机遇大于挑战的环境。目前，国际金融危机尚未见底，对实体经济的影响正在进一步加深，其严重后果还会进一步显现，世界经济增长将明显减速。受外部环境影响，我国经济运行中的困难增多，经济下行压力加大。在这样的国际国内宏观形势下，我们面临着最严峻的考验，保增长促发展的任务十分艰巨。在看到形势严峻、困难增多的同时，我们更应该看到我市加快发展的有利条件。尽管我国经济发展面临着来自国际国内严重困难和严峻挑战，但外部冲击没有改变经济发展的基本面和长期发展趋势，我国发展的重要战略机遇期仍然存在；中央已经并将继续出台一系列扩大内需、促进经济增长的强有力政策措施，加大对民生工程、基础设施和生态环境建设等重点领域的投入；省委、省政府为应对当前形势，也出台了一系列促进经济平稳较快增长的具体措施。这些都为我市扩大投资、调整结构、增强后劲、改善民生创造了良好的外部环境。随着沿海地区经济加速转型，产业向中西部地区转移的趋势加快，为我市积极参与泛长三角区域分工与合作，高层次、大范围承接产业转移带来了难得的机遇。合芜蚌自主创新综合配套改革试验区的启动建设，为我们提供了新的发展动力和空间。通过近些年来全市上下的共同努力，一批重大产业项目全面实施，我市经济综合实力不断增强，加之良好的区位优势，为我们今后的发展奠定了坚实的基础。特别是人民群众中蕴含着无限的创造力和全市人民过上更加美好生活的新期待，必将进一步激发全社会的创业热情，为推进经济社会发展注入强大动力。面对机遇和挑战，我们必须振奋精神，知难而进，以积极的态度应对挑战，以主动的作为抢抓机遇，形成攻坚克难的合力和韧劲，不分心、不走神、不争论、不松劲，全力以赴抓好各项工作落实，努力保持经济平稳较快发展。

    今年市政府工作的总体要求是：全面贯彻党的十七大和十七届三中全会精神，以邓小平理论和"三个代表"重要思想为指导，认真学习实践科学发展观，以保增长为首要任务，深入实施工业强市战略，巩固强化农业基础，促进消费需求持续增长，狠抓招商引资和项目投资，推进自主创新，调整经济结构，深化改革开放，保障改善民生，统筹发展各项社会事业，努力促进全市经济社会又好又快发展。

    今年经济社会发展的主要预期目标为：地区生产总值增长10%以上，一、二、三产分别增长5%、12.5%和10%，规模以上工业增加值增长15%以上；财政总收入增长11%，其中地方财政收入增长13%；固定资产投资增长20%以上；利用境外资金增长15%，引进市外资金增长30%；社会消费品零售总额增长12%；进出口总额增长11%；城镇居民人均可支配收入增长6%；农民人均纯收入增长6%；城镇登记失业率、人口自然增长率、居民消费价格涨幅、化学需氧量排放及二氧化硫排放均控制在省下达的目标以内；万元地区生产总值能耗降低4.5%。

    以上预期目标，是在全面分析宏观形势，综合考虑各种因素的基础上确定的，总体上说是积极可行的，通过努力也是能够实现的。在实际执行中，要在提高质量和效益的前提下，力争发展的更快一些。完成上述目标任务，需要我们把握好以下几个原则：一是必须坚持发展第一要务，牢牢扭住经济建设这个中心，继续解放思想，增强机遇意识，坚定发展信心，聚精会神搞建设，一心一意谋发展。二是必须坚持自主创新，把推进自主创新作为加快经济结构调整和发展方式转变的战略支点，加快推进创新型城市建设，深化改革开放，增强发展的活力和动力。三是必须坚持实施工业强市战略，做到扩大规模与提升质效并举，产业集聚与优化布局互动，加快推进新型工业化进程。四是必须坚持把项目投资和招商引资作为经济工作的主要抓手，继续推动招商引资升温加压，千方百计扩大投资规模，增强发展后劲。五是必须坚持统筹协调发展，着力保障和改善民生，全面发展各项社会事业，推进城乡一体化建设，努力构建和谐蚌埠。

    三、采取扎实有效措施，努力保持经济平稳较快发展

    (一)强力推进工业发展，着力提高工业化水平。以促进结构调整为主线，以项目建设为抓手，以培育骨干企业为重点，努力提高工业经济增长的质量和效益。一是加快产业结构调整升级步伐。进一步做大做强优势产业，培育打造新兴产业，改造提升传统产业。重点支持装备制造及零部件、精细化工、电子信息、光伏、生物质、新材料六大产业加快发展，力争六大产业完成投资100亿元、产值350亿元。加快食品、纺织等传统产业技术改造，提高产品层次和附加值。二是扶持企业做大做强。继续实施大企业、大集团培育计划，推进国有大中型企业主业整合和战略性重组，实施技术标准化战略和品牌带动战略，提高产品质量和市场竞争力，重点支持丰原生化、天宝集团等大企业做大做强。积极实施专、精、特、新中小企业培育计划，加大政策、项目扶持力度，全年新增规模以上企业80户。三是狠抓工业项目建设。重点抓好200个千万元以上工业项目，完成工业投资120亿元。落实项目责任制，确保昊业年产3000万只高效滤清器及总成、华益年产400万片抗反射膜玻璃等50个项目竣工投产，促成柳工年产5000台起重机研发基地、双环年产2亿只精密薄膜电阻器等50个项目开工建设。组织谋划编排一批工业项目，新增项目储备150亿元。四是大力推进园区建设。进一步明确园区产业定位，促进园区产业集聚，推进昊方机电、浙泰不锈钢等产业集群发展。继续推进高新区申报国家级开发区和扩区工作，加快市区及乡镇工业集中区建设。推进光伏产业园建设，形成100兆瓦非晶硅薄膜太阳能电池生产能力。加快玻璃新材料科技产业园、精细化工园等园区建设步伐。着手建设汽车工业园起步区。继续做好电子信息产业园、粮食产业园、船舶工业园的谋划和前期工作。积极推动八一股份公司老厂区等市区企业"退市进园"，促进企业向园区集聚。五是强化为企业服务工作。开展"企业服务年"活动，认真落实好向部分园区、企业、项目派驻服务人员制度，尽心竭力为企业排忧解难。加强政银企合作力度，支持引导银行机构加大信贷投入。建立上市融资奖励专项资金，鼓励企业上市融资、发行债券融资，扩大直接融资规模。建立中小企业信用担保和贷款风险补偿基金，努力缓解中小企业融资难问题。通过综合协调煤电油运、减轻企业负担等手段，切实解决企业市场销售困难、成本压力过大等实际问题。引导企业生产更多适销对路的产品，推动企业加强管理和创新，提高经济效益和市场竞争力。

    (二)大力发展农村生产力，促进"三农"工作全面发展。以增加农民收入为核心，以发展现代农业为方向，以创新农村工作机制为动力，以改善农村生产生活条件为保障，巩固和扩大农业农村良好发展势头。推进现代农业建设。继续实施水稻、小麦、玉米三大粮食生产行动，稳定面积，主攻单产，提升品质。加快农业产业化经营步伐，重点扶持60家龙头企业做大做强，新增各类农民专业合作社40个，培育特色产业示范村40个。加强农业标准化建设，大力发展优质畜禽、水产品等特色产业，新增市级标准化养殖小区20个。提升农业科技应用水平，加快良种和先进适用技术示范与推广，新增高效农业面积10万亩，积极推进天河省级高效生态农业项目建设。狠抓农产品质量安全，突出源头治理，新增农产品认证8个。改善农村生产生活条件。实施好樵子涧水库除险加固等七项重点治淮工程建设，解决15万农村人口饮水安全问题，新增农村户用沼气2000口，成片造林1.1万亩，建设绿色长廊30公里。开展村容整治活动，实施农村清洁工程。千方百计增加农民收入。加强农民负担监管，落实各项强农惠农政策，实施政策性农业保险，抓好农村劳动力转移培训"阳光工程"，加快建设农民工返乡创业园，努力拓宽农民增收渠道。推进农村改革创新。稳定和完善农村基本经营制度，规范土地承包经营权流转，发展适度规模经营，在各县开展土地复垦整理工作试点。支持县域经济发展。把发展工业作为促进县域经济发展的最重要工作来抓，鼓励和支持发展各具特色的县域工业，促进农村流通业和现代服务业加快发展，壮大县域经济实力。

    (三)加快发展第三产业，努力保持消费需求持续增长。支持发展生产性服务业。推进姜桥物流园区、光彩物流中心、丰原铁路专用线建设，大力发展第三方物流。实施水运振兴工程，加快蚌埠新港二期、蚌埠危险品码头、固镇煤炭专用码头建设。进一步提升海关、检验检疫等口岸服务功能。加强金融服务与监管，优化金融生态环境，鼓励金融创新，增强金融支撑经济发展的能力。继续开展小额贷款公司试点，加快推进农村合作银行、村镇银行组建工作。积极培育会展、证券期货、信息咨询等服务业。大力发展生活性服务业。继续改造提升商贸流通业，加快合百新商厦、商之都、沃尔玛购物中心、宝龙城市广场、华运蔬菜批发市场皖北配送工程等重点项目建设进度，推进淮河路步行街区、经济开发区商业街区和淮上区商贸经济圈建设。重点培育钢材、家居、古玩玉器市场，开工建设安徽农机大市场、生猪交易市场。继续推进"万村千乡"市场工程和"新网工程"，启动"家电下乡"工程，拉动农村消费。加快发展旅游经济，加大双墩古文化遗址、涂山风景区、天河风景区等旅游资源开发力度，推进龙子湖旅游风景区基础设施建设。强化政策引导，鼓励住房消费，促进房地产业平稳健康发展。加快医疗、社区服务等消费产业发展。

    (四)狠抓项目投资和招商引资工作，切实增强发展后劲。继续实施投资带动战略，扩大投资规模，优化投资结构，提高投资质量。加大项目和资金争取力度。抢抓国家扩大内需的机遇，紧紧围绕保障性安居工程、民生工程、自主创新和结构调整等投向，扎实做好项目前期工作，积极争取中央预算内投资和国家长期建设国债，大力争取国家和省专项建设资金，力争落实各类补助资金3亿元以上。加快重点项目建设。继续推进"3461"行动计划，重点项目投资比重占固定资产投资40%以上。建成国电蚌埠电厂一期2号机组、北泰汽配工业园制动器等项目，加快日元贷款城市水环境治理、京沪高铁蚌埠段等续建项目，开工建设滤清器产业园、高铁新区基础设施等重点项目，力争开工建设大庆路淮河公路桥、徐明高速公路蚌埠段、蚌淮高速延伸段，推进国电蚌埠电厂二期、天宝乘用车等项目前期工作。继续提升项目服务水平。加强项目调度和督查，优化项目工作流程，改善外部施工环境，保障项目建设有效推进。强化项目储备工作，不断充实项目库，新增项目储备规模300亿元以上。

    进一步加大招商引资工作力度。强势推动招商引资工作，坚定不移地把招商引资工作作为经济工作的重中之重，继续加压升温，切实做到精力再集中、力量再加强、措施再加大，力争在大招商、招大商、招群商方面取得突破。不断创新招商方式。大力开展小分队招商、以商招商等多种形式招商，拓宽引资渠道。主攻招商重点。瞄准江苏、浙江、上海、福建等沿海重点区域，抢抓长三角和珠三角产业转移的机遇，打造产业承接平台，吸引更多的项目落户我市。开展丰富有效的招商活动。积极参加全国性、区域性的各类经贸活动，重点举办30次以上面向长三角、珠三角等发达地区投资说明会及产业对接活动。完善考核奖惩机制。健全定期召开调度会制度，适时通报责任单位招商情况，积极督促引进资金及时到位。改进考核督查制度，加强对招商引资实效性考核和督查，严格兑现奖惩。

    四、推进自主创新综合配套改革，努力建设创新型城市

    (一)全面推进自主创新，加强创新能力建设。努力打造创新型产业基地。启动国家级玻璃新材料高新技术产业基地和省级汽车零部件、光伏、生物质高新技术产业基地申报认定工作，创建国家大学科技园，加快规划建设生物医药工业园。积极提升企业创新能力。支持企业设立技术中心，新增省级企业技术中心3家，争创国家级企业技术中心，启动市级企业技术中心认定工作。新认定高新技术企业6家、专利优势企业4家、申报各类科技计划项目200项。培育中国名牌产品1个、争创安徽名牌3个。整合检测资源，筹建检验检测集中区，推进5家企业和科研院所参与制定国家行业标准、技术标准和产品标准。加大自主创新投入力度。设立自主创新综合配套改革试验区财政专项资金，建立多元化的科技创新投融资体制。加强知识产权保护，建成国家专利技术展示交易中心，确保全年专利申请量达550件。

    (二)扩大改革开放，提升对外合作与交流水平。继续深化重点领域改革，激发创新活力。健全国有资产监管体系，推进国有企业改革，完成丰原集团、华皖碳纤维等国有企业改制工作。不断深化公共财政制度改革，开展财政支出绩效评价监督试点，强化预算监督管理。进一步完善市对区财政体制。深化土地管理制度改革，积极申报国家集约节约用地试点。加大"空心村"治理力度，加快闲置土地清理处置工作。深化人事分配制度改革，探索收入分配激励机制，抓好创新型人才培养和引进。继续推进科技、教育、文化、卫生等管理体制改革。

    全方位扩大对外开放，增强发展动力。用足用好外贸促进政策，实施科技兴贸和品牌兴贸战略，推进国家级科技兴贸出口基地建设，全力支持柠檬酸、滤清器、非晶硅太阳能电池等自主品牌产品出口，不断开拓国际市场，努力保持出口稳定增长。大力推动安徽水利等15家企业"走出去"，增强我市外经企业利用"两个市场、两种资源"做大做强的能力。主动参与泛长三角区域发展分工，突出承接产业转移。积极参与"两淮一蚌"城市群建设，实现区域经济协调发展。扩大友好城市合作，拓展对外交往领域。

    (三)继续推动全民创业，大力发展非公有制经济。全面落实鼓励全民创业的政策措施，加快各类创业基地建设，完善创业服务体系，努力形成全民竞相创业的生动局面。鼓励和支持非公有制企业提升管理水平、加快品牌培育、参与国有经济战略性重组和产业链配套，增强竞争能力和发展后劲。力争新增个体工商户9000户、私营企业1500家，非公有制经济占国民经济比重达到56%以上。

    五、高度关注民生，加快和谐蚌埠建设

    (一)围绕建设淮畔名城，强化城市建设与管理。加强规划编制工作。加快编制城市分区规划和淮河南岸、城市地下空间等各类专项规划，完善旧城改造地区、近期发展地区控制性详细规划。推进土地利用总体规划修编，因地制宜地搞好乡镇和村庄规划。完善城市功能。完成中荣街、中山街等道路改造和体育路东段打通工程，建成朝阳北路、淮上大道，实施城市南出口、凤阳路西段、南山路中段改造工程，适时对胜利路进行综合提升改造，推进黄山大道、延安南路、龙子湖西岸地区路网工程建设。开工建设八里沟、南施家、新船塘排涝站。建成张公山桥北公园，建设张公岛花卉苗木基地，启动政务区南部区域开发。开工建设陶山变、青年变等输变电工程。强力推进城市管理。深化城市管理试点，加强城市管理数字化建设，积极探索城市管理新机制。深入开展文明创建活动，重点加强占道经营、户外广告、交通秩序等专项整治，让整个城市逐步净起来、亮起来、美起来。

    (二)围绕优化生态环境，加强节能减排工作。进一步加强环境保护工作。抓好重点企业污染治理，规范各污水处理厂的运行与管理，提高第三污水处理厂运行负荷，第二污水处理厂建成投入运行，开工建设城南污水处理厂。继续开展工业固废、大气、噪声及农业面源和畜禽养殖污染等防治工作，实施医疗固废集中处置。加强林地、湿地保护，重点推进四方湖、沱湖湿地、三汊河省级湿地公园规划建设。切实加强资源节约利用。鼓励和支持企业采用节能环保新设备、新工艺、新技术进行节能减排技术改造，加快发展清洁能源，突出抓好重点领域、重点产品、重点企业节能工作。严格实行节能减排目标责任制，大中型建设项目环评执行率达95%以上，规模以上工业企业万元增加值综合能耗下降5.5%。

    (三)围绕提高文明程度，推进社会事业建设。加快制定市区中小学布局规划，加强薄弱学校改造，提高中小学教学质量，扩大省示范高中办学规模，大力发展职业教育，提升高等教育水平。发展社区卫生、农村卫生事业，完善公共卫生服务体系，推进市疾控中心选址新建工作。加强文化遗产保护，筹办双墩刻划符号国际研讨会。弘扬花鼓灯文化，大力发展公益性文化事业。提高体育设施建设水平，广泛开展全民健身活动，做好省十二届运动会备战工作。加快推进新档案馆、二中新校区、三院新院区建设工作。稳定低生育水平，提高出生人口素质。启动有线电视数字化整体转换和移动电视广播工作。大力弘扬和实践蚌埠城市精神，实施公民道德建设工程，加强未成年人思想道德建设。整顿和规范市场秩序，依法严厉打击传销。积极筹建皖北食品药品检验中心，加强以食品药品和餐饮卫生安全为重点的市场监管。严格安全生产责任制，坚决防止重特大安全事故。深化"平安蚌埠"建设，完善社会治安防控体系，积极预防和妥善处理群体性事件。加强和改进信访工作，完善信访机制，坚持依法治访，逐步建立良好的信访秩序。扎实做好国防动员、民兵预备役、人防工作，深入开展双拥共建活动，繁荣发展社会科学，加强民族、宗教和对台等方面的工作。

    (四)围绕改善人民生活，着力解决群众关注的民生问题。积极筹措资金，组织实施好省28项民生工程。继续改善群众生活条件，提高城乡居民最低生活保障标准，扩大农村低保覆盖面，完善城镇未参保集体企业退休人员基本生活费保障机制；继续提高群众健康水平，实行市辖区新农合工作市级统筹管理，进一步完善重大传染病病人医疗救治和艾滋病病人生活救助保障机制，加快城乡卫生基础设施建设，完成4所乡镇卫生院、111所村卫生室和27个社区卫生服务中心(站)标准化建设；继续保障学生受教育权利，免除城乡义务教育阶段学生学杂费，提高农村贫困寄宿生生活费补助标准和农村义务教育阶段中小学生均公用经费标准，建立健全高校和中职学校家庭经济困难学生资助制度；继续改善群众居住条件，对人均住房建筑面积低于13平方米的城市低收入住房困难家庭应保尽保，加快实施31个棚户区改造和城中村改造，推进36.7万平方米政策性保障住房建设；继续解决农村群众出行不便问题，加快推进农村公路、渡改桥工程建设；落实好"农家书屋"工程、乡镇综合文化站建设、农民体育健身工程、农村五保供养服务机构建设、重度残疾人生活救助、提高妇女儿童健康水平等11项新增民生工程。

    在实施民生工程的同时，按照"量力而行、尽力而为"的原则，今年还将为群众再办10件实事：实施积极的就业政策，新增2.9万个城镇就业岗位；进一步健全被征地农民养老保险制度，覆盖面达到85%以上；正式启动失业保险市级统筹；新增3所市区学校接纳进城务工农民子女接受义务教育；统一市属医院门诊病历手册，实行看病就医"一本通"；改造20条城市主次道路，并完成路灯配套；新建改造10座、维修20座城市公厕；新购置公交车辆40台，新增及延伸公交线路3条；建设占地1.3万平方米的公交枢纽站和信息化调度中心；启动市博物馆、艺术中心等建设。各级政府要不断增强公共服务能力，真正把民生工程建设成民心工程，努力促进社会和谐发展。

    六、加强政府自身建设，着力提高行政效能

    2009年，国际国内宏观经济环境将更加严峻，发展任务将更加繁重而艰巨，政府工作面临巨大压力和重大考验。我们必须振奋精神，创新思路，知难而进，切实增强领导科学发展和驾驭复杂局面的本领，以更加昂扬的斗志、更加务实的作风、更加有为的工作，从容应对各种挑战，不断开创蚌埠跨越发展、争先崛起的新局面。

    要解放思想，增强本领。进一步解放思想，大力倡导和弘扬敢闯敢试、敢作敢为的开拓精神，创新管理理念，完善工作方法，创造性地开展工作。以建设创新服务型政府为目标，深化行政管理体制改革，健全部门之间协调配合机制，着力解决职能交叉、权责脱节、政出多门的问题。加强学习和调查研究，提高服务发展、解决问题的能力和水平，增强工作的预见性、主动性和创新性。加快应急管理体系建设，全面提高保障公共安全和处置突发事件的能力。

    要依法行政，接受监督。牢固树立依法行政理念，自觉接受人大的法律监督和政协的民主监督，认真办理人大代表议案、建议和政协提案，广泛接受群众监督和舆论监督。建立健全重大决策征求意见、听证、合法性审查和责任追究等制度，提升决策科学化、民主化水平。认真落实政府信息公开条例，扩大政务公开范围。继续办好政风行风热线、政风行风面对面和政民连线等栏目，畅通群众监督诉求渠道。加强行政执法队伍建设，强化行政执法责任追究，规范行政执法行为。

    要强化执行，转变作风。增强大局意识，加强团结协作，进一步提高政策执行力和工作落实力。继续深化机关效能建设，打通"中梗阻"，扫除"拦路障"，确保政令畅通、令行禁止。强化"一站式"服务，推进"两集中、两到位"改革，推行审批服务标准化建设，贯通市、县行政服务网络体系，力求行政审批服务高标准、高效率、快节奏。建立政府绩效管理制度，对重大决策部署、重点项目、民生工程等落实和完成情况进行综合绩效考评，加大对政府部门履行职责的检查、监督与考核力度，形成层层抓落实、事事做到位、件件有着落的工作机制。要弘扬求真务实、真抓实干的作风，以作风的积极转变促进各项工作的有效推进。坚持深入基层、深入实际，努力为群众谋好事、办实事、解难题。大力精简会议、文件，清理规范检查、评比、达标活动，把精力集中到谋发展、抓落实上。

    要廉洁从政，艰苦奋斗。加强反腐倡廉教育，提高廉洁自律能力，严格执行廉洁从政的各项规定。建立健全教育、制度、监督并重的惩治和预防腐败体系，从源头上预防和治理腐败。强化行政监察和审计监督，严肃查处违法违纪案件，坚决纠正损害企业和群众合法权益的行为。牢记"两个务必"，大力发扬艰苦奋斗、勤俭节约的优良传统，建设节约型机关。政府和政府各部门必须有过紧日子的思想准备，加强增收节支，降低行政成本，节约有效资源，把剩下来的钱用在改善民生上，用在支持企业发展和自主创新上，用在加快城市基础设施建设上。加强对公务员的培训、管理、监督和交流轮岗力度，努力建设一支高素质的公务员队伍。

    各位代表，我们正处在一个充满挑战和竞争的时代，也处在一个呼唤创新和崛起的时代。发展前景无限美好，使命崇高而神圣，责任光荣而重大。让我们以邓小平理论和"三个代表"重要思想为指导，认真贯彻落实科学发展观，在中共蚌埠市委的坚强领导下，坚持好字优先、能快则快不动摇，坚持创新创业、攻坚破难不停步，坚持富民惠民、促进和谐不松懈，振奋精神，昂扬斗志，开拓进取，求实务实，努力推动蚌埠经济社会又好又快发展，以优异的成绩迎接中华人民共和国诞辰60周年！
